# Applying the UTAUT2 framework to patients’ attitudes toward healthcare task shifting with artificial intelligence

**DOI:** 10.1186/s12913-024-10861-z

**Published:** 2024-04-11

**Authors:** Weiting Huang, Wen Chong Ong, Mark Kei Fong Wong, Eddie Yin Kwee Ng, Tracy Koh, Chanchal Chandramouli, Choon Ta Ng, Yoran Hummel, Feiqiong Huang, Carolyn Su Ping Lam, Jasper Tromp

**Affiliations:** 1 National Heart Centre Singapore, 5 Hospital Drive, Singapore, 169609 Singapore; 2 Duke-NUS Medical School, Singapore, Singapore; 3 Saw Swee Hock School of Public Health, National University of Singapore, National University Health System Singapore, Singapore, Singapore; 4 Us2.ai, Singapore, Singapore; 5 School of Mechanical and Aerospace Engineering, Nanyang Technological University, Singapore, Singapore; 6 National Healthcare Group Polyclinics, Singapore, Singapore

**Keywords:** Healthcare artificial intelligence, Machine learning, Task shifting, Point-of-care ultrasound, Echocardiography, Technology acceptance, Patient attitudes

## Abstract

**Background:**

Increasing patient loads, healthcare inflation and ageing population have put pressure on the healthcare system. Artificial intelligence and machine learning innovations can aid in task shifting to help healthcare systems remain efficient and cost effective. To gain an understanding of patients’ acceptance toward such task shifting with the aid of AI, this study adapted the Unified Theory of Acceptance and Use of Technology 2 (UTAUT2), looking at performance and effort expectancy, facilitating conditions, social influence, hedonic motivation and behavioural intention.

**Methods:**

This was a cross-sectional study which took place between September 2021 to June 2022 at the National Heart Centre, Singapore. One hundred patients, aged ≥ 21 years with at least one heart failure symptom (pedal oedema, New York Heart Association II-III effort limitation, orthopnoea, breathlessness), who presented to the cardiac imaging laboratory for physician-ordered clinical echocardiogram, underwent both echocardiogram by skilled sonographers and the experience of echocardiogram by a novice guided by AI technologies. They were then given a survey which looked at the above-mentioned constructs using the UTAUT2 framework.

**Results:**

Significant, direct, and positive effects of all constructs on the behavioral intention of accepting the AI-novice combination were found. Facilitating conditions, hedonic motivation and performance expectancy were the top 3 constructs. The analysis of the moderating variables, age, gender and education levels, found no impact on behavioral intention.

**Conclusions:**

These results are important for stakeholders and changemakers such as policymakers, governments, physicians, and insurance companies, as they design adoption strategies to ensure successful patient engagement by focusing on factors affecting the facilitating conditions, hedonic motivation and performance expectancy for AI technologies used in healthcare task shifting.

**Supplementary Information:**

The online version contains supplementary material available at 10.1186/s12913-024-10861-z.

## Background

Healthcare providers worldwide are constantly challenged to achieve the ‘quadruple aim’ in care delivery: Improving population health, enhancing patient experience, reducing healthcare worker burnout and dissatisfaction, and reducing costs [1, 2]. There has been great progress in healthcare artificial intelligence (AI) and machine learning (ML) in the recent years [3–6], which hold promise.

Healthcare AI technologies has seen tremendous adoption especially in the medical imaging field [7–9]. AI’s performance is shown to match or exceed human experts in studies involving computed tomography [10], chest X-ray classification [11], ophthalmology [12, 13], oncology [14, 15] and dermatology [16]. Such advances demonstrate how AI can refine diagnostic accuracy and improve patient outcomes, while using healthcare resources more efficiency. However, there are still challenges and limitations in the data quality, ethical issues, regulatory frameworks, and user acceptance of AI technologies [17].

In our main study, a novice layperson with no prior sonography knowledge was trained and equipped with a point-of-care(POC) ultrasound device [18], which had real-time AI image guidance on probe repositioning to capture better image quality. We also used a United States Food and Drug Agency-approved deep learning-based software [19], which automatically analyses the acquired images and provides cardiac measurements such as the left ventricular ejection fraction (LVEF). We named this workflow the AI-novice combination. Our study showed that the AI-novice combination, compared with trained sonographer-performed and cardiologist-interpreted echocardiogram studies, was able to achieve an area under the curve (AUC) of 0.880 (95%CI 0.802, 0.958) in detecting patients with left ventricular dysfunction. Furthermore, the AI-novice combination had a median absolute deviation from the reference standard LVEF at 6.03%, which is comparable to human inter-reader variability [20].

Apart from the AI-novice combination’s accuracy, we wanted to study the patients’ attitudes and hence usage intention, given that AI taskshifting is a new model of care. There are several theories which look at technology usage intention behaviours. For our study, we chose the unified theory of acceptance and use of technology (UTAUT) and its extended version (UTAUT2) because it was the most comprehensive [21, 22]. Among the UTAUT2 framework’s constructs, we examined the relationships between performance expectancy, effort expectancy, social influence, facilitating conditions, hedonic motivation and behavioral intention. We did not include questions on price and trust in the framework as the cost and reliability of the new AI-novice combination had not been established at the point of study. Also, the questions on habit pertain more towards voluntary consumerism, hence it was not applicable to a physician-ordered investigation. Additionally, the UTAUT2 has been one of the more important and versatile theoretical models used to predict consumer technology use and its effectiveness has been proven in different cultural backgrounds [23].

Prior studies largely looked at healthcare workers’ adoption intention of AI-assisted diagnosis and treatment [24]. In contrast, our study looks at the patients’ perspective, using the UTAUT2 framework. This has not been studied comprehensively, to our knowledge. Since patients are key stakeholders in new care pathways, this study will contribute to better understanding of factors influencing patient usage intention, therefore reduce barriers and facilitate implementation of AI-enabled taskshifting.

### Study hypothesis and theoretical foundation

#### Hypothesis 1

Performance expectancy influences the intention to accept task shifting of specialized scans to AI-novice combination.

Performance expectancy describes the perception that using the technology will benefit the patient and is therefore tied to the perception of usefulness [21]. In most studies, performance expectancy significantly influenced behavioural intention and was also often the strongest predictor [25].

#### Hypothesis 2

Effort expectancy influences the intention to accept task shifting of specialized scans to AI-novice combination.

Effort expectancy describes the expected ease of using the technology [21].

#### Hypothesis 3

Social influence affects the intention to accept task shifting of specialized scans to AI-novice combination.

Social influence is “the degree to which an individual perceives that important others believe he or she should use the new system” [21].

#### Hypothesis 4

Facilitating conditions influence the intention to accept task shifting of specialized scans to AI-novice combination.

Facilitating conditions refer to the perceptions “of the resources and support available to perform a behavior” [21].

#### Hypothesis 5

Hedonic motivation influences the intention to accept task shifting of specialized scans to AI-novice combination.

Hedonic motivation refers to gratification (fun, pleasure, and enjoyment) associated with using technology [21]. It is considered as the most important theoretical addition to the UTAUT2 as it shifted the focus from extrinsic motivation of an organization to intrinsic motivation of consumers toward usage of technologies [26].

Behavioural intention refers to the degree in which a person will perform a specified action, in this case accepting the task shifting of specialized scan to the AI-novice combination. It is the variable that responds to the above hypotheses.

## Methods

### Sample and data collection

In this cross-sectional study, patients aged ≥ 21 years with at least one heart failure symptom (pedal oedema, New York Heart Association (NYHA) II-III effort limitation, orthopnoea, breathlessness), were prospectively included. Consecutive patients presenting to the cardiac imaging laboratory at the National Heart Centre Singapore for physician-ordered clinical echocardiogram for investigation or follow-up for heart failure were approached. All patients underwent standard cart-based sonographer-performed, cardiologist-reported echocardiogram as per clinical practice. For the study, patients then underwent an additional experience of a novice-performed AI-supported POC echocardiogram on the same day for comparison. After the AI-novice combination echocardiogram was performed, a questionnaire was administered individually to each patient. The questionnaire comprised two portions; the first collected demographic information, while the second focused on the key constructs of performance and effort expectancy, facilitating conditions, social influence, hedonic motivation and behavioural intention. The second part of the questionnaire was adapted from the original version developed by Venkatesh et al. [21], see supplementary file 1.

#### Ethics approvals

Ethics approvals were obtained from the local institutional review committee for this study, and this study conformed to the ethical guidelines in the Declaration of Helsinki.

### Measurement and data analysis

The items measuring performance expectancy, effort expectancy, social influence, facilitating conditions, hedonic motivation and behavioral intention were adapted from Venkatesh et al. [21] to suit our study. We modelled our questionnaire as closely as possible to the original questionnaire by Venkatesh et al. [21], excluding only questions that did not suit our context. These items were measured using 5-point Likert scales anchored by 1(strongly disagree) and 5(strongly agree). Table 1 displays the finalized items with the mean scores of each question.

**Table 1:** Extended unified theory of acceptance and use of technology survey questionnaire and mean scores

Item	Mean score ± SD
PE1. The AI-novice echocardiogram will give an accurate risk stratification of my cardiac condition.	3.78 ± 0.73
PE2. The AI-novice echocardiogram will enable a more efficient clinic consult	3.87 ± 0.74
PE3. I trust the results of the AI-novice echocardiogram.	3.71 ± 0.75
PE4. The results of the AI-novice echocardiogram will give me reassurance when it is normal	3.96 ± 0.65
PE5. I trust the healthcare staff performing the AI-novice echocardiogram screening on me	4.14 ± 0.58
EE1. It would not take the healthcare staff long to learn how to use AI-novice echocardiogram	3.79 ± 0.79
EE2. The AI-novice echocardiogram would be easy to learn	3.80 ± 0.79
EE3. It would be easy for the healthcare staff to become skillful at using the AI-novice echocardiogram even when they do not have ultrasound training	3.79 ± 0.78
EE4. Performing the AI-novice echocardiogram on me should be easy	3.91 ± 0.66
SI1. People who are important to me think that I should use the AI-novice echocardiogram before clinic visit	3.67 ± 0.72
SI2. People whose opinions I value would like me to use the AI-novice echocardiogram	3.69 ± 0.70
FC1. The healthcare workers have the knowledge necessary to use the AI-novice echocardiogram	4.06 ± 0.64
FC2. The AI-novice echocardiogram device is similar to other screening tools such as the electrocardiogram, retinal eye screening etc.	3.73 ± 0.87
FC3. The novice has the resources necessary to perform the AI-novice echocardiogram.	3.97 ± 0.61
HM1. The process of screening via the AI-novice echocardiogram is enjoyable	3.65 ± 0.70
HM2 The process of screening via the AI-novice echocardiogram is fun	3.59 ± 0.88
BI1. I intend to be a frequent user of AI-enabled screening devices	3.60 ± 0.81
BI2. I intend to continue using AI-screening devices in future	3.72 ± 0.72

The analysis process was divided into two parts. As the questionnaire was adapted, the measurement quality was rigorously checked for reliability and validity. Only when the quality of the model was confirmed, the structural model was analyzed and interpreted.

Composite reliability (CR) and Cronbach’s *α* are used to evaluate internal consistency reliability. Both CR and Cronbach’s *α* values are recommended to be greater than 0.7 [27].

Convergent validity is supported if all the standardized item loadings are greater than 0.70, and if the average variance extracted (AVE) values for every construct exceed 0.5, the threshold value recommended by Fornell and Larcker [28]. Items which did not meet the criteria of > 0.70 were dropped from the final partial least squares structural equation modelling (PLS-SEM).

Discriminant validity is achieved if both AVE estimates of a given pair of constructs are greater than the square of the construct correlation [29].

PLS-SEM was used to explain causal relationships among constructs [30], as it could support both exploratory and confirmatory relationships [31–34]. This is suitable in this context as the questionnaire has been adapted from the original to suit our study. This regression analysis also allows for smaller sample sizes and permits estimation of models using ordinal scale data [35]. Additionally, prior studies of UTAUT2 with adapted questionnaires also used PLS-SEM as the preferred method [36–38]. PLS-SEM evaluations are comprised of an outer model (measurement model) and an inner model (structural model).

The structural model was validated using a nonparametric bootstrap procedure to test the statistical significance of path coefficients with 5,000 bootstrap samples [27].

All analyses were performed using Stata *statistical software* (version 14.0, Stata Corporation, College Station, Texas, USA). All statistical analyses were conducted at the significance level of 0.05, and all tests were two-tailed whenever appropriate.

## Results

The sample characteristics are presented in Table 2. Of the respondents, 56% were male, the average age was 61.2 years, and 33% of respondents received tertiary or higher education.

**Table 2:** Baseline demographics of patients

Demographics	(*n* = 100)
Age (years)	61.2 ± 15.0
Male	56 (56%)
Education	
No formal education Primary Secondary Tertiary	2 (2%) 10 (10%) 55 (55%) 33 (33%)
Race	
*Chinese* *Malay* *Indian* *Others*	75 (75%) 8 (8%) 13 (13%) 4 (4%)
Hypertension	55 (55%)
Diabetes Mellitus	27 (27%)
Dyslipidemia	64 (64%)
Systolic Blood Pressure (mmHg)	130 ± 19
Diastolic Blood Pressure (mmHg)	75 ± 8
Current Smoker	13 (13%)
History of Heart Failure	40 (40%)
History of Coronary Artery Disease	29 (29%)
Symptom of Leg Swelling	23 (23%)
Symptoms of Orthopnea	12 (12%)
Symptoms of Breathlessness	
*None* *On Exertion* *At Rest* *Anytime*	39 (39%) 48 (48%) 1 (1%) 12 (12%)
New York Heart Association Class	
I II III IV	39 (39%) 50 (50%) 6 (6%) 5 (5%)

Internal consistency reliability, convergent and discriminant reliability.

The internal consistency reliabilities for all constructs were greater than the threshold value (i.e. *α* ≥ 0.7; CR ≥ 0.7). However, the item loadings for Question 5 of Performance Expectancy and Question 4 of Effort Expectancy were < 0.70; hence these 2 items were excluded from the PLS-SEM. The other items had met criteria for being statistically significant (*p* < 0.001) and greater than 0.70 (see Table 3).

**Table 3:** Convergent validity and construct reliability

Item	Standardized Item Loadings
Performance Expectancy (α = 0.82, CR = 0.83)	
PE1. The AI-novice echocardiogram will give an accurate risk stratification of my cardiac condition.	0.84
PE2. The AI-novice echocardiogram will enable a more efficient clinic consult	0.82
PE3. I trust the results of the AI-novice echocardiogram.	0.75
PE4. The results of the AI-novice echocardiogram will give me reassurance when it is normal	0.70
PE5. I trust the healthcare staff performing the AI-novice echocardiogram screening on me	0.68
Effort Expectancy (α = 0.75, CR = 0.75)	
EE1. It would not take the healthcare staff long to learn how to use AI-novice echocardiogram	0.75
EE2. The AI-novice echocardiogram would be easy to learn	0.83
EE3. It would be easy for the healthcare staff to become skillful at using the AI-novice echocardiogram even when they do not have ultrasound training	0.78
EE4. Performing the AI-novice echocardiogram on me should be easy	0.67
Social Influence (α = 0.89, CR = 0.89)	
SI1. People who are important to me think that I should use the AI-novice echocardiogram before clinic visit	0.94
SI2. People whose opinions I value would like me to use the AI-novice echocardiogram	0.94
Facilitating Conditions (α = 0.77, 0.79)	
FC1. The healthcare workers have the knowledge necessary to use the AI-novice echocardiogram	0.82
FC2. The AI-novice echocardiogram device is similar to other screening tools such as the electrocardiogram, retinal eye screening etc.	0.79
FC3. The novice has the resources necessary to perform the AI-novice echocardiogram.	0.90
Hedonic Motivation (α = 0.82, CR = 0.84)	
HM1. The process of screening via the AI-novice echocardiogram is enjoyable	0.70
HM2 The process of screening via the AI-novice echocardiogram is fun	0.70
Behavioral Intention (α = 0.89, CR = 0.89)	
BI1. I intend to be a frequent user of AI-enabled screening devices	0.81
BI2. I intend to continue using AI-screening devices in future	0.81

After excluding Question 5 of Performance Expectancy and Question 4 of Effort Expectancy, the AVE values for all constructs were greater than 0.5. Thus, convergent validity was supported. The square of the correlation coefficients was smaller than the corresponding square roots of the AVE estimates for all pairs of constructs. Therefore, discriminant validity was achieved (see Table 4).

**Table 4:** Correlation matrix and descriptive statistics of the constructs

Construct	Performance Expectancy	Effort Expectancy	Social Influence	Facilitating Conditions	Hedonic Motivation	Behavioural Intention
Performance Expectancy	1.000 (0.533)					
Effort Expectancy	0.197	1.000 (0.517)				
Social Influence	0.189	0.159	1.000 (0.806)			
Facilitating Conditions	0.187	0.225	0.098	1.000 (0.591)		
Hedonic Motivation	0.277	0.176	0.164	0.236	1.000 (0.710)	
Behavioural Intention	0.326	0.097	0.299	0.285	0.254	1.000 (0.820)

The values on the diagonal (in parantheses) are the square root of average variance extracted (AVE) estimates

### The structural model

**Fig. 1:**
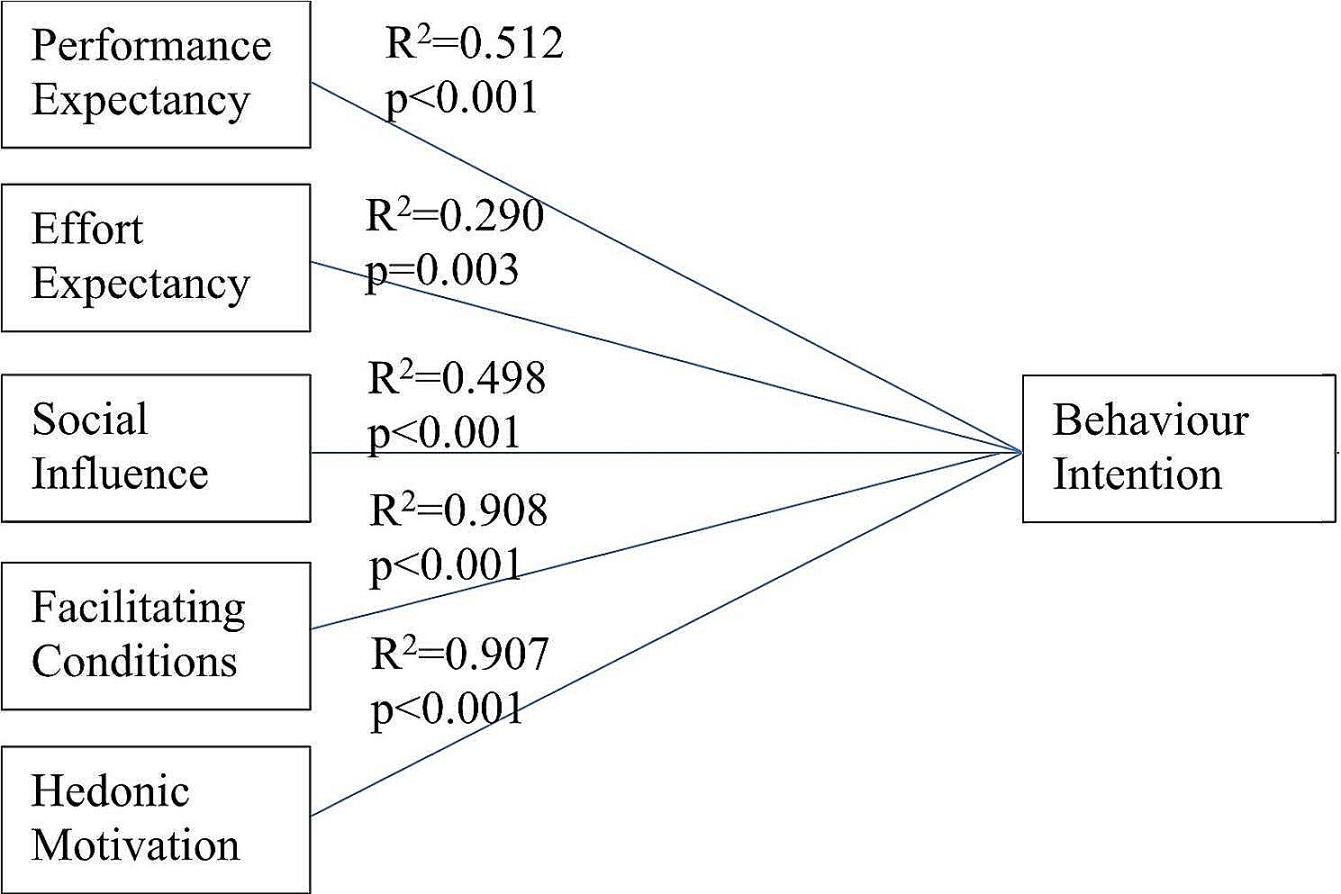
Resulting path coefficients after bootstrapping analysis for constructs affecting behvioural intention to accept task shifting of specialized scans to AI-novice combination in the extended UTAUT2 model

The resulting path coefficients are depicted in Fig. 1, after bootstrapping analysis.

The results revealed that the extended UTAUT2 model could explain 85% of the variance in behavioural intention. All the hypothesized variables showed significant relationship to behavioral intention. Facilitating conditions, hedonic motivation and performance expectancy showed the strongest relationship to behavioural intention.

Patient factors such as age (*p* = 0.181), education level(*p* = 0.218) and gender(*p* = 0.776) did not significantly affect behavioural intention.

## Discussion

Task shifting of specialized studies is one key area where AI is helping to revolutionize healthcare. Examples include interpreting medical imaging, such as chest x-rays [39], and implementing nationwide screening programs, such as retinal imaging [40]. While the medical community focuses on the accuracy and theoretical foundations of the AI, studies have shown that despite comparable or even superior accuracy of AI, patients have found it challenging to accept AI in medical care [41, 42]. Hence in our study, apart from determining the accuracy of the AI-novice combination, we also sought to study the motivators and barriers from the patient’s perspective toward acceptance of such new models of care.

The UTAUT2 suggests that actual use of any technology is affected by one’s behavioural intention to use it. It proposes that four main constructs affect behavioural intention: usage, intention—performance expectancy, effort expectancy, social influence and facilitating conditions. It extends three more constructs (hedonic motivation, price value and habit) in its framework. The UTAUT2 provides the highest explanatory power of all standard acceptance models. Knowing these underlying important constructs will help organizations improve their technology implementation processes [21, 22].

Our study showed that all four main constructs showed a significant relationship with behavioural intention, influencing 85.8% of variance to behavioural intention. The top 3 constructs influencing behavioural intention in our study were facilitating conditions, hedonic motivation, and performance expectancy. While medical performance expectancy was the most important construct in many other studies that focused on healthcare professionals [25], facilitating conditions may exert stronger influence from the patients’ perspective [43, 44]. This does not mean that performance expectancy, which is the perception that using the technology will benefit the patient, is less important; rather, in a complex network like the healthcare system, facilitating conditions play an important and significant role in access. Hedonic motivation, which is the fun and pleasure derived from using the technology, represents an affective component of the UTAUT2 [26] and looks at the user’s intrinsic motivation [45, 46] rather than the extrinsic motivation from the four main constructs. Our study shows that hedonic motivation is a significant factor in behavioural intention. Therefore, making the process fun and pleasurable is important towards patient adoption [47]. Facilitating conditions and hedonic motivation had a strong correlation (*r* = 0.992, *p*-value < 0.001) in our study. This intuitively suggests that having the resources to improve access itself also improves overall patient experience and enjoyment, placing further importance on facilitating conditions in the successful implementation of this new pathway.

Age, gender and education levels were not significant modifiers of behavioural intention. This was congruent with prior studies on health apps, where age, gender and education levels were weakly correlated with behavioural intention [36].

Prior studies of UTAUT2 in healthcare largely looked at patients; acceptance toward digital health applications [36, 37, 48, 49] and have largely found that hedonic motivation, social influence and habit, rather than utilitarian factors predicted usage intention. As the nature of digital health applications are different from AI-assisted taskshifting and diagnosis, we felt that it was important to apply the UTAUT2 framework in this new model of care. In the area of AI-assisted diagnosis, from the healthcare workers’ perspective, Cheng et al. [50] demonstrated that performance and effort expectancy directly influenced usage intention. Our study showed that patients have a different considerations, suggesting that it is important to look at factors affecting different stakeholders to ensure successful implementation.

The sustainability of new pathways and health programs requires understanding the implementation process and factors that impact adoption. The UTAUT2 is one of the most comprehensive theoretical frameworks developed to investigate factors related to future usage intention [47]. When detailing the implementation process, understanding these factors will allow changemakers to focus more on certain aspects, such as facilitating conditions, hedonic motivation and performance expectancy. Our study is the first to look at patients’ attitudes toward task shifting using AI in healthcare, using the UTAUT2 theory. This provides important information for the implementation of such pathways in future.

### Limitations

Our study has several limitations. First, our study is based in Singapore, which has high levels of access to digital technologies. Based on the Singapore Digital Society Report 2023 [51], 97% of Singaporeans own a smartphone. The study also found that 65% of Singaporeans are keen to try out new digital technologies. This limits the generalizability of our study’s findings to settings that are less advantaged technologically, but it may still be used to make future predictions in these settings. Furthermore, we did not control for patients’ digital literacy, technology acceptance and attitudes towards AI in general in our study, which may be potential confounders for our study’s findings. In our study, we had chosen to omit items with item loadings < 0.70 for convergent validity, after ensuring that each construct had at least two questions for measurement. We recognize that by omitting these items, especially borderline cases, can result in missing out important information that can be measured by the question. Also, our study’s design offered patients the experience of both the standard cardiac sonography and AI-novice workflow for comparison on the same day. While the study team attempted to distinguish the two experiences by explanation and conducting the AI-novice workflow only after the standard echocardiogram as per protocol, we acknowledge that patients may not be fully able to distinguish their experiences and perceptions for each method clearly and this may limit the accuracy of our study’s findings.

## Conclusion

This study shows that all constructs proposed by the UTAUT2 model are essential for patients’ successful acceptance of AI-enabled novice-performed echocardiogram. These results are important for stakeholders and changemakers such as policymakers, governments, physicians, and insurance companies, as they design adoption strategies to ensure successful patient engagement by focusing on factors affecting the facilitating conditions, hedonic motivation and performance expectancy for AI technologies used in healthcare task shifting. Future studies can look into the practical design of adoption strategies based on these key factors and to evaluate their impact on patients’ acceptance of similar AI technologies.

### Electronic supplementary material

Below is the link to the electronic supplementary material.

Supplementary Material 1

## Data Availability

The datasets used and/or analysed during the current study are available from the corresponding author on reasonable request.

## Abbreviations

AI: Artificial intelligence
AUC: Area under the curve
AVE: Average variance extracted
CR: Composite reliability
DL: Deep learning
ML: Machine learning
LVER: Left ventricular ejection fraction
PLS: SEM–partial least squares structural equation modelling
UTAUT2: Unified theory of acceptance and use of technology, extended version
