# Supplementary material for: Applying the UTAUT2 framework to patients’ attitudes toward healthcare task shifting with artificial intelligence
Source: BMC Health Serv Res. 2024 Apr 11;24:455. doi: 10.1186/s12913-024-10861-z (PMC11007870; doi:10.1186/s12913-024-10861-z)
Supplement: Supplementary file 1 — Supplementary Material 1 [file 12913_2024_10861_MOESM1_ESM.docx]

| Item | Strong Disagree | Disagree | Neutral | Agree | Strongly Agree |
| --- | --- | --- | --- | --- | --- |
| *Performance expectancy* (*PE*) the degree an Artificial Intelligence-enabled, handheld echocardiogram will provide benefits to patients during their clinic consult | | | | | |
| PE1. The AI-novice echocardiogram will give an accurate risk stratification of my cardiac condition. |  |  |  |  |  |
| PE2. The AI-novice echocardiogram will enable a more efficient clinic consult |  |  |  |  |  |
| PE3. I trust the results of the AI-novice echocardiogram. |  |  |  |  |  |
| PE4. The results of the AI-novice echocardiogram will give me reassurance when it is normal |  |  |  |  |  |
| PE5. I trust the healthcare staff performing the AI-novice echocardiogram screening on me |  |  |  |  |  |
| *Effort expectancy* (*EE*) the degree of ease associated with *AI-enabled handheld echocardiogram* | | | | | |
| EE1. It would not take the healthcare staff long to learn how to use AI-novice echocardiogram |  |  |  |  |  |
| EE2. The AI-novice echocardiogram would be easy to learn |  |  |  |  |  |
| EE3. It would be easy for the healthcare staff to become skillful at using the AI-novice echocardiogram even when they do not have ultrasound training |  |  |  |  |  |
| EE4. Performing the AI-novice echocardiogram on me should be easy |  |  |  |  |  |
| *Social influence* (*SI*) the extent to which consumers perceive that important others (e.g., family and friends) believe that I should allow AI-enabled handheld echocardiogram screening during my initial clinic visit | | | | | |
| SI1. People who are important to me think that I should use the AI-novice echocardiogram before clinic visit |  |  |  |  |  |
| SI2. People whose opinions I value would like me to use the AI-novice echocardiogram |  |  |  |  |  |
| *Facilitating conditions* (*FC*) consumer’s perceptions of the resources and support available to use the AI-enabled echocardiogram |  |  |  |  |  |
| FC1. The healthcare workers have the knowledge necessary to use the AI-novice echocardiogram |  |  |  |  |  |
| FC2. The AI-novice echocardiogram device is similar to other screening tools such as the electrocardiogram, retinal eye screening etc |  |  |  |  |  |
| FC3. The novice has the resources necessary to perform the AI-novice echocardiogram. |  |  |  |  |  |
| *Hedonic motivation* (*HM*) the fun or pleasure derived from having the AI-enabled echocardiogram performed on the patient | | | | | |
| HM1. The process of screening via the AI-novice echocardiogram is enjoyable |  |  |  |  |  |
| HM2 The process of screening via the AI-novice echocardiogram is fun |  |  |  |  |  |
| *Behavioral intention* (*BI*) a person’s perceived likelihood or subjective probability that he or she will engage in a given behavior | | | | | |
| BI1. I intend to be a frequent user of AI-enabled screening devices |  |  |  |  |  |
| BI2. I intend to continue using AI-screening devices in future |  |  |  |  |  |
